# Supplementary material for: Biosynthesis of Poly-(3-hydroxybutyrate) under the Control of an Anaerobically Induced Promoter by Recombinant Escherichia coli from Sucrose
Source: Molecules. 2022 Jan 4;27(1):294. doi: 10.3390/molecules27010294 (PMC8746831; doi:10.3390/molecules27010294)
Supplement: Supplementary file 1 [file molecules-27-00294-s001.zip › molecules-1532139-supplementary.pdf]

Supplementary Materials

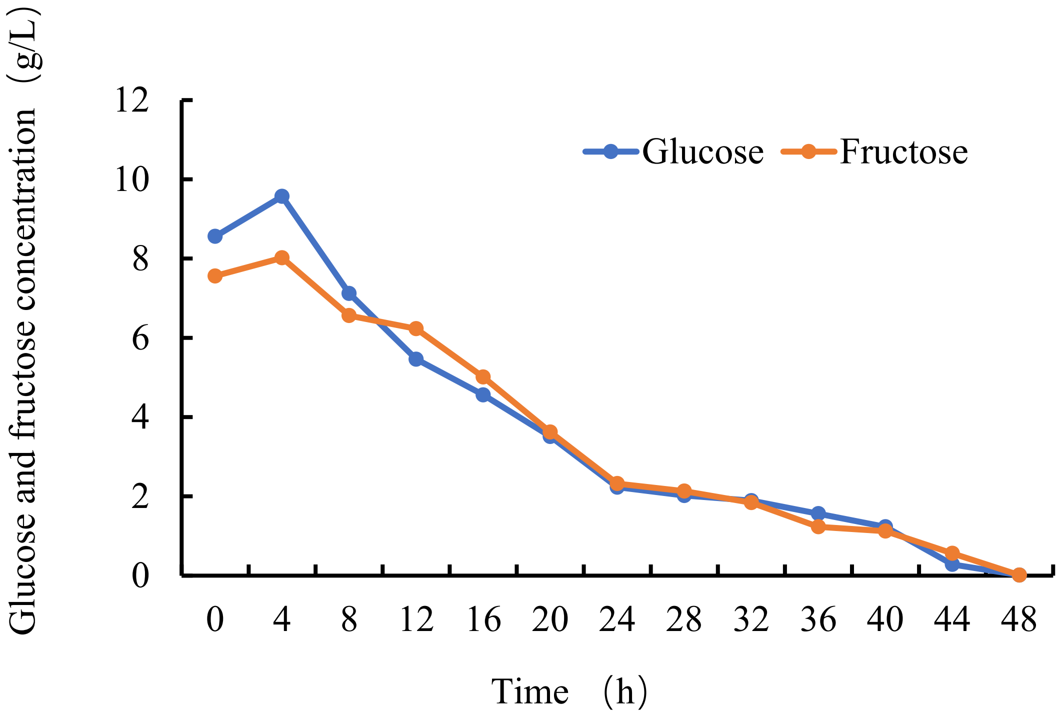

Figure S1. Concentrations of glucose and fructose in batch fermentation for PHB production.
